# Can a power law improve prediction of pain recovery trajectory?

**DOI:** 10.1097/PR9.0000000000000657

**Published:** 2018-06-13

**Authors:** George C. Hartmann, Steven Z. George

**Affiliations:** a Strategy and Innovation Group, Xerox Corporate Research and Technology, Webster, NY, USA [retired]; b Musculoskeletal Research, Duke Clinical Research Institute, Durham, NC, USA; c Clinical Research, Department of Orthopaedic Surgery, Duke University, Durham, NC, USA

**Keywords:** Chronic pain, Power law, Complex systems

## Abstract

**Introduction:**

Chronic pain results from complex interactions of different body systems. Time-dependent power laws have been used in physics, biology, and social sciences to identify when predictable output arises from complex systems. Power laws have been used successfully to study nervous system processing for memory, but there has been limited application of a power law describing pain recovery.

**Objective:**

We investigated whether power laws can be used to characterize pain recovery trajectories.

**Methods:**

This review consists of empirical examples for an individual with complex regional pain syndrome and prediction of 12-month pain recovery outcomes in a cohort of patients seeking physical therapy for musculoskeletal pain. For each example, mathematical power-law models were fitted to the data.

**Results:**

This review demonstrated how a time-dependent power law could be used to refine outcome prediction, offer alternate ways to define chronicity, and improve methods for imputing missing data.

**Conclusion:**

The overall goal of this review was to introduce new conceptual direction to improve understanding of chronic pain development using mathematical approaches successful for other complex systems. Therefore, the primary conclusions are meant to be hypothesis generating only. Future research will determine whether time-dependent power laws have a meaningful role in improving strategies for predicting pain outcomes.

## 1. Introduction

Chronic pain is characterized as nervous system condition resulting from dynamic and complex interactions among biological, behavioral, environmental, and societal factors.^12^ Complex systems science approaches have been advocated for advancing understanding of movement variability in physical therapy^5^ and for better management of other chronic noncommunicable diseases such as obesity.^24^ It is beyond the purpose of this review to describe different aspects of complex systems science, thoroughly described in other sources.^2,3^ Instead, we focus on time-dependent power laws because of their use across diverse fields to reveal underlying regularity in complex systems.^20^ Specifically, we will consider if power laws can be used to characterize pain trajectories and enable better understanding of when pain becomes chronic.

Stevens investigated the relation between the magnitude of a physical stimulus and the subjective magnitude of the sensation, finding that often these obey power laws.^23^ Albert and Barabási^1^ reviewed how time-dependent power laws emerge from different networks under a variety of modeling assumptions; time-dependent power laws have been derived in a wide range of settings including physics, biology, and social sciences.^8^ Specifically, there is precedent for power-law phenomena involving nervous system processing. Early application of power laws included investigations of memory. The “forgetting curve” was first measured by Hermann Ebbinghaus in 1885 and later replicated by Murre and Dros.^19^ This was modeled^18^ to show that human memory diminishes according to a time-dependent power law. Power laws have also been used to define the size distribution of neuronal avalanches in cortical networks.^15^

Considering similarities between memory and pain^13^—can power laws be used to improve understanding of pain trajectories? This is a viable question for the field to consider now that it is accepted that chronic pain results from complex and dynamic interactions between different systems. One reason a power law is relevant for pain is that updated versions of biopsychosocial models emphasize how determinants of health result from a dynamic system, unfolding over time at an individual level.^16^ Time-dependent power laws are well suited to characterize such complexity, even on an individual scale.^20^ Exploration of power law in pain is also justified as an opportunity to improve on prediction of outcomes. Prediction models using linear regression approaches have not enabled large improvements in accuracy, and it is difficult to apply models validated in groups of patients for prediction of an individual outcome.^4^ This is especially true if the prediction time is a free parameter because many existing prediction models are based on fixed follow-up time points. If time-dependent power laws are relevant, a patient or provider could anticipate when future progress may occur, instead of just being able to anticipate if progress may occur.

Another reason to explore power laws for pain is the potential for imputing missing outcomes data, a common problem with longitudinal databases or registries.^17,21^ The question of how to account for missing data will be important to address as more of these databases accumulate specific to pain. Commonly used imputation methods (eg, last value forward and mean value substitution) are known to bias results.^14^ A power-law function could be a viable alternative method for imputing missing data because it has the appropriate curvature for interpolating adjacent data points.

Therefore, the purpose of this review is to investigate whether trajectories for pain recovery can be represented by time-dependent power laws. This review will be completed using 2 empirical examples. The first is a case study of complex regional pain syndrome (CRPS) as a proof-of-concept demonstration; the second is a cohort of patients seeking care for musculoskeletal pain to determine accuracy of future pain-intensity ratings.

## 2. Methods

### 2.1. Application of power law to individual pain recovery

In March 2015, a 75-year-old man (G.C.H.) fell, causing an intertrochanteric fracture of the left leg that was repaired surgically. Complex regional pain syndrome developed postsurgically and was treated with medication and exercise. After 24 months, the patient continued to have pain and used his pain-medication dose history to reconstruct his pain trajectory and predict future improvements. Doctors prescribed oxycodone for 3 months followed by gabapentine for 6 months. Over time, pain intensity decreased, and medication dose was correspondingly reduced.

There is little evidence supporting the effectiveness of long-term opioid use for chronic pain conditions^7^ and wide variability in prescribing patterns for orthopedic conditions.^22^ As a result, in every day clinical practice, opioid dosages are titrated to an individual response.^9^ In this case example, as the pain situation improved, the dosage was gradually decreased over many weeks. The individual patient experience was that when the no-medication-pain was high intensity, the pain-medication dose had to be increased to reduce the after-medication-pain to a tolerable level, denoted by the patient as “residual pain.” To estimate the time dependence of the patient's no-medication-pain intensity level, we assumed that the no-medication-pain was approximately proportional to the individual dose. When switching medications, the patient's perception was that 1 mg oxycodone had about the same effect as 300 mg gabapentine.

**Figure 1.**
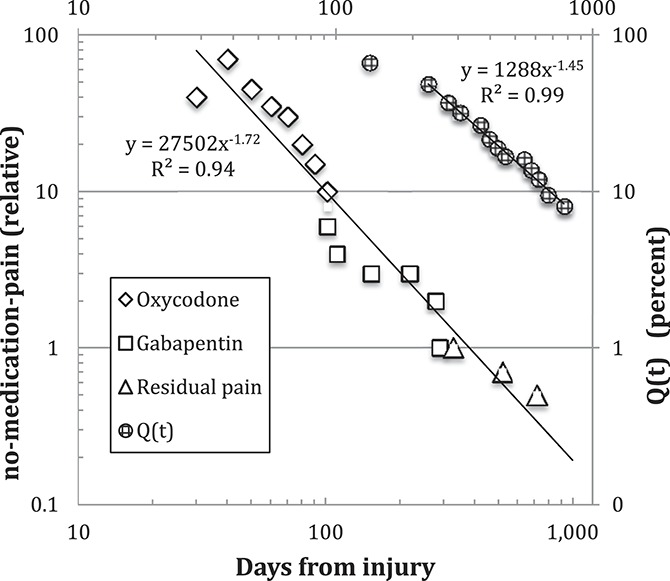
Log–log plot of no-medication CRPS pain (left scale) and muscle strength gap Q(t) (right scale) vs time. CRPS, complex regional pain syndrome.

After pain medication was discontinued, the remaining pain gradually diminished another 50% over the following year. The trajectory of no-medication-pain intensity is shown in Figure 1. A time-dependent power law of the form
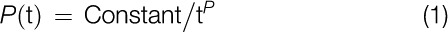
was fitted, providing an indication of how the no-medication-pain intensity decreased over time and might decrease in the future. These findings motivated us to further explore the utility of a power-law model using data from a cohort study.

The pain trajectory in Figure 1 raises the question of how the no-medication-pain scale, which spans almost 2 decades, maps to the numerical rating scale (NRS) pain scale. One possibility is that “no-medication-pain” is related to the NRS by a geometric series, specifically 2^N^, where N represents the NRS pain level. The step ratio was estimated by comparing 2 widely separated points of the pain record: the NRS was judged to be 8 in month 2, masked by 70 mg daily oxycodone; the residual NRS level was 2 at month 24, with no medication. Because there are 6 steps from level 8 to 2, the step ratio is approximately 2. We emphasize that this relationship is incidental to the applicability of a power-law model and that this n = 1 case served only as a conceptual study.

Figure 1 also shows the recovery of left leg strength, atrophied due to CRPS. Physical recovery was followed by tracking the average S(t) of 7 strength tests performed on gym machines (expressed as left/right leg strength ratios) and plotting the strength gap Q(t) = 1 − S(t) against time. This strength gap decline also follows a time-dependent power law.

### 2.2. Application of power law for pain recovery in a musculoskeletal pain cohort

Pain-intensity ratings from the Optimal Screening for Prediction of Referral and Outcome (OSPRO) validation cohort were used for this analysis.^10^ This cohort consisted of 440 individuals seeking physical therapy with primary complaint of neck, shoulder, knee, or shoulder pain. Patients could have chronic or acute pain complaints, as well as have postoperative pain.

**Figure 2.**
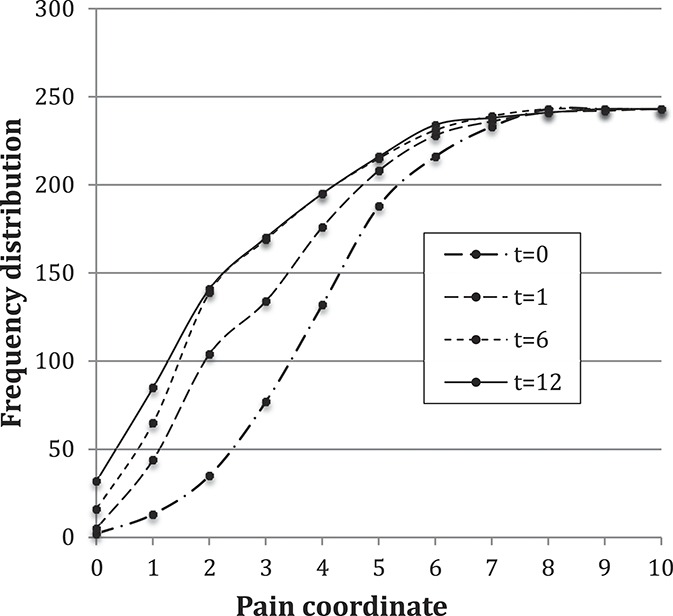
Cumulative frequency distribution of the average-NRS-pain-intensity. NRS, numerical rating scale.

Pain-intensity scores on a 0 to 10 NRS were collected at initial physical therapy consultation and then at 4 weeks, 6 months, and 12 months later. At each session, the NRS was used to rate current, best and worst pain intensity. These scores were averaged to create an average-NRS-pain-intensity rating. There were 243 patient records that reported pain intensity for every time point. Figure 2 displays the cumulative frequency distribution of all 243 records. Overall, the distributions are broad with an approximately constant SD at each time (average value 1.9).

**Figure 3.**
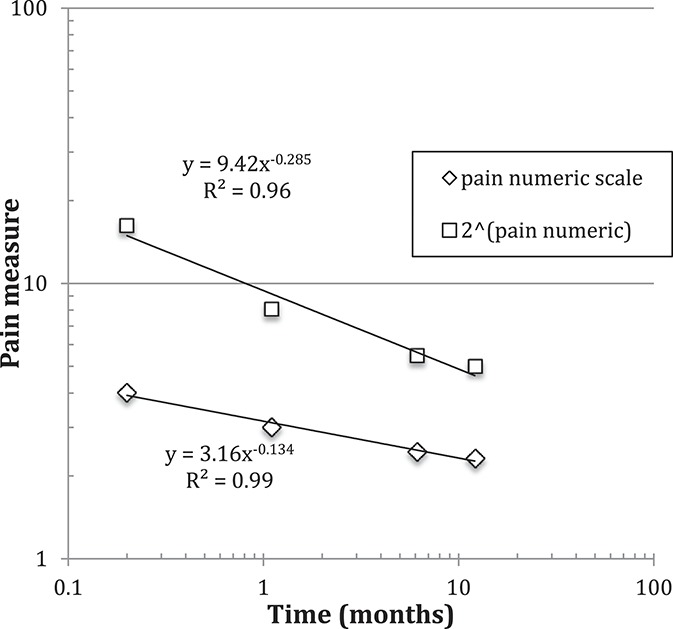
Time dependence of the average-NRS-pain-intensity, on a log–log plot. NRS, numerical rating scale.

The mean value of the average-NRS-pain-intensity data was plotted against time in Figure 3, and a power law was fitted. We examined whether an alternative model, a simple time-dependent exponential, could provide a faithful representation of the data. A key difference between these 2 models is that the power-law model is more skewed, with a long tail that declines much more slowly with time compared with an exponential model. The goodness-of-fit was gauged using χ^2^. For the power-law model, we found χ^2^ = 1.0 and 2.6 for average-NRS-pain and pain-geometric scales, respectively, whereas for the alternative exponential model, χ^2^ = 22 and 25 for the same respective scales. This difference in goodness-of-fit was an order of magnitude more favorable for the power-law model, suggesting that it better represents the time dependence of the data compared with an exponential model.

Next, we examined how accurately 12-month outcomes could be predicted from individual pain records. Two calculation approaches were investigated. In the first approach, each of the 243 patient records was separately fitted by a power law with 1 free parameter. This approach yielded 243 values of the prefactor, whereas the exponent was constrained to a single value. To assess prediction accuracy of the first calculation approach, we made 3 projections for each patient record using data from 3 combinations of time marks: I (0 and 6 months); II (0, 1, and 6 months); and III (0 and 1 month). In the second calculation approach, each patient record was separately fitted by a power law with 2 free parameters yielding 243 values for both the exponent and prefactor. The accuracy of the second calculation approach was assessed with projection IV (0, 1, and 6 months).

For the first calculation approach, the value of the free parameter (prefactor) for every patient record was computed at each time mark, 
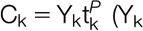
 (Y_k_ represents the pain data), then averaged across the time marks. The pain predicted at a future time T was computed using 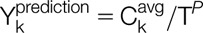. A single value of the exponent was used (*P* = 0.3) determined from projection IV. For the second calculation approach, the values of 2 free parameters (prefactor and exponent) were found by linear regression for every patient record.

**Figure 4.**
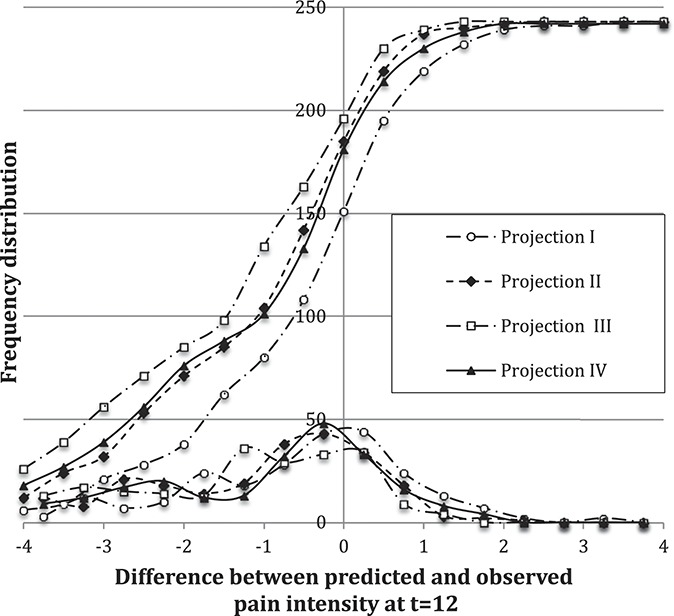
Frequency distributions of deviation between projected and observed 12-month pain scores using a power law with a fixed exponent (projection I, II, and III) and a fitted exponent (projection IV). The 4 curves in the upper group are cumulative frequency distributions; the 4 curves in the lower group are frequency distributions.

**Figure 5.**
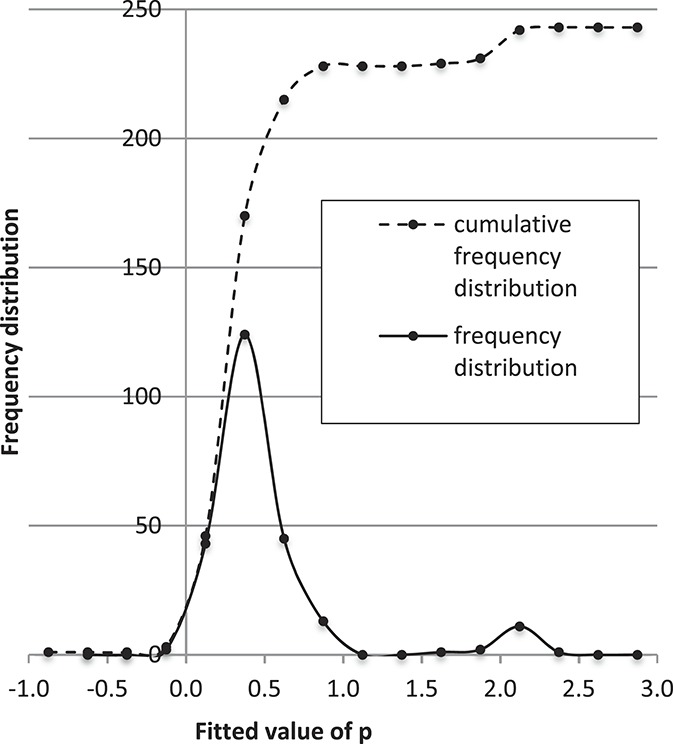
Frequency distribution of the power-law exponent determined by linear regression against individual patient records for projection IV.

To quantify the predictive accuracy, Figure 4 presents the frequency distribution of the difference between the observed and predicted 12-month pain values, 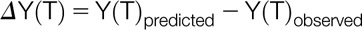. These frequency distributions provided the accuracy metric reported in Table 1, which estimates the percentage of time that the power-law prediction at 12 months is within a tolerance band ±1, 1.5, or 2 units on the average-NRS-pain scale. Figure 5 shows that the frequency distribution of *P* for projection-method IV is narrow, with a mean value of about *P* = 0.3. The narrow shape suggests that the data are well represented by a power law.

**Table 1:**
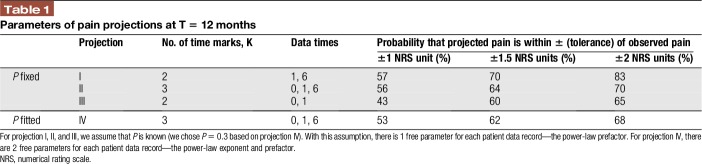
Parameters of pain projections at T = 12 months

Comparing projections II and IV, Table 1 and Figure 4 show that these are essentially identical. Projection IV (2 degrees of freedom) might be expected to be somewhat better than projection II (1 degree of freedom). They are essentially identical because both use the power-law shape, and the power-law exponent values are similar. A marginally less accurate result is obtained whenever data at time mark t = 0 are included. The cumulative distributions in Figure 2 suggest that the data scatter at each time mark is similar. However, the cumulative distribution of the scatter of slope values transitioning from t = 0 to t = 1 is 6 times as broad as the scatter for other time transitions. Thus, the cross-correlations within each patient record change with time and include stronger correlations at later time points, suggesting why the inclusion of t = 0 data marginally (but consistently) reduced the projection accuracy.

**Figure 6.**
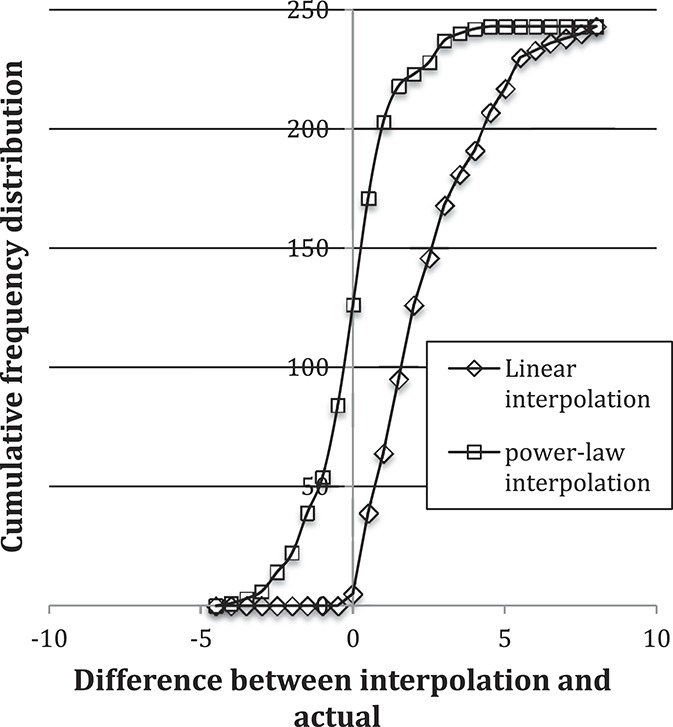
Cumulative frequency distributions that compare linear and power-law interpolation methods for data missing at time mark t = 6, using data at time marks t = 1 and t = 12.

Finally, we performed a preliminary examination of using a power law for imputing missing data by omitting time mark t = 6 and using t = 1 and t = 12 data to impute t = 6. We compared 2 interpolation methods as part of this preliminary examination. The first was linear interpolation with 2 free parameters (slope and intercept) and the second was power-law interpolation with 2 free parameters (prefactor and exponent). The cumulative frequency distributions of the difference between the actual t = 6 data measurement and the imputed data are displayed in Figure 6. Linear interpolation gives a systematic discrepancy of about 2 NRS pain units, together with a markedly asymmetric distribution around the midpoint, whereas power-law projection was centered solidly at zero with a symmetric distribution. Linear interpolation systematically overestimates the value because the data trend is concave upward. This preliminary investigation suggests that power law may be useful for imputing missing data.

## 3. Discussion

This review described how time-dependent power law may have application for better understanding of musculoskeletal pain recovery trajectories. The included examples provide conceptual direction for considering whether using complex system approaches, such as power laws, can be used to better describe pain recovery, similar to what has been performed in movement variability^5^ and to explore new management models in obesity.^24^ Pain recovery modeled by a time-dependent power law may be a better mathematical match for updated biopsychosocial models that emphasize the dynamic, individual influence on health outcomes.^16^ Finally, this review demonstrated that there may be potential for power-law functions to be considered as an alternative to existing methods of missing data imputation.

A physical model for a pain trajectory power law and its derivation is provided in the Appendix. If a power-law model faithfully replicates the pain trajectory, it can be used to estimate the time required for a given pain intensity level to reduce by half. From Equation 1, the ratio M of the 2 times is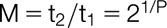. As an example, for the CRPS case study *P* = 1.7, so M = 1.5. At day 30, a 50% pain reduction is anticipated by 30M = 45 days (another 15 days). At month 24, 50% pain reduction occurs by 24M = 36 months (another 12 months). If future pain reduction continues in this fashion, one can set realistic expectations for time of recovery. This approach is quite different from a prediction which focuses on the probability of an event occurring at a fixed time. Furthermore, additional research in this area is needed to determine whether commonly used pain scales (eg, NRS) operate in a scalar or geometric manner when the goal is predicting pain reduction.

In the cohort study, we demonstrated 2 ways that a power-law model can be used to project recovery from care seeking for musculoskeletal pain. One method is to monitor recovery progress over a time interval of modest duration to confirm that the symptoms are indeed following a power law, and use this time sequence to estimate the power-law exponent. Then, Equation 1 can then be used to project future improvements. A second method is to use an a priori value of *P* determined from previous work. Progress can be projected straightaway using Equation 1, extrapolating from early patient pain assessments.

**Figure 7.**
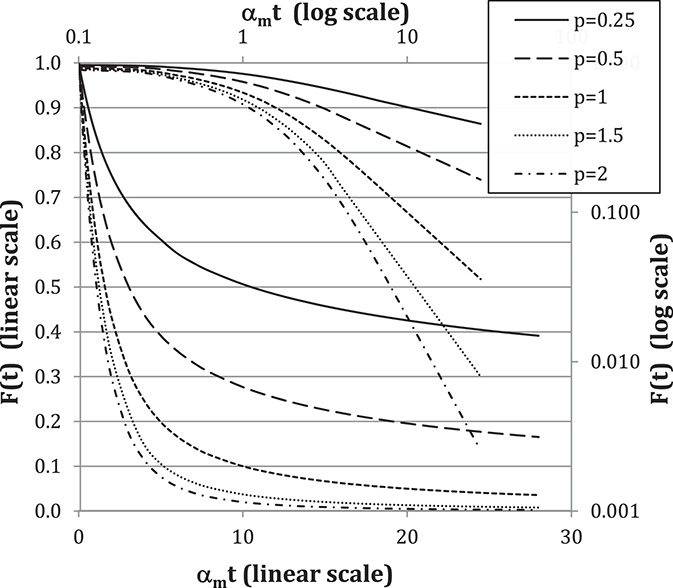
Power-law model F(t) calculated using Equation 6. The time-series plotted on logarithmic scales (right and upper) asymptotically become straight lines when t is large. The same time-series plotted on linear scales (left and lower) show long tails that diminish very slowly with time, especially if *P* is small.

In the cohort example, the values of the exponent *P* were quite small, resulting in a large M value and a correspondingly large time interval for 50% decrease to occur. Consider for example, a patient with a small exponent value *P* = 0.4 and an NRS-pain-intensity rating of 2 at 1 year; the M value projects that 5 additional years will be required for a 50% decrease. This may not be such an unrealistic prediction given that pain improvement often plateaus after the first 6 months. Within the framework of a power law, if the exponent *P* is small, progress can appear to be very slow as illustrated by Figure 7. This may be a different way to characterize “chronic pain.” It would simply be a consequence of a small value of *P*. Conversely, rapidly improving pain would manifest with larger *P* values. The advantages to this approach would be that the recovery trajectory of the individual is used to define a chronic state, instead of relying on definitions that may not universally apply to a given population.

In moving forward, several points need to be addressed. First, cohorts with more than 3 time points should be used to further explore whether time-dependent power law is a good fit for describing recovery trajectories. From a predictive modeling perspective, we demonstrated in the Appendix that a time-dependent power law^20^ can emerge from situations where exponential decay of many ensemble elements is aggregated. The power-law exponent value depends on the shape of the distribution of the exponential decay time constants. Therefore, future work remains to propose a physical model for what biological factors might determine this shape and to confirm that a power law is the best fit for this shape in comparison with other functions. In our investigation, there were a small percentage of patients who experienced a worsening of pain included together with the majority of patients experiencing a lessening of pain. This was an intentionally conservative approach for this review; future work remains to determine whether patients who have worsening pain reports should be modeled by other nonlinear time-dependent functions.

Furthermore, an important question remains regarding how pain medication or other pain interventions received during recovery might modify the power-law pain trajectory. Future research could focus on determining if power-laws could be used to make comparisons of treatment effectiveness that are more sensitive than current statistical methods.

Finally, there is potential for power-law functions to be used to impute missing data from longitudinal cohort studies. This is an important area for future study because reviews of the literature suggest that suboptimal methods are often used in registries^21^ or cohort studies.^14,17^ Specifically, comparisons of power-law interpolation to a current state-of-art approach, such as multiple imputation,^11^ might provide meaningful information on how best to account for the inevitable problem of missing data.

## 4. Conclusions

This review demonstrated how time-dependent power laws can be applied to pain recovery trajectories for a case study of CRPS and a cohort of patients with musculoskeletal pain. This review was intended to be hypothesis generating and to introduce new conceptual direction for pain by using mathematical approaches successful in describing other complex systems. Future research will determine whether time-dependent power laws have a meaningful role in predicting pain outcomes, determining treatment effectiveness, or accounting for missing data.

## Disclosures

The authors have no conflict of interest to declare.

S.Z. George received NIH support while writing this manuscript (NAIMS award AR055899 and NCCIH award AT009790).

## Appendix A. Derivation of Power-law Model

A power law can arise from the summation of many elements, weighted by their frequency of occurrence in a specific way—with each element decaying exponentially with time, e^−αt^, where α is the decay rate. Conceptually, these decaying elements may represent the healing trajectories of injured nerve or muscle fibers. The starting point is to express the ensemble decay as the sum of many individual exponential decays having a range of decay rates between 2 limits 0 and α_m_,

Here, f(α) is a frequency distribution function for the parameter α, normalized so that . The normalization assures that F(0) = 1.

The value of the power-law exponent *P* turns out to depend on the unknown shape of the distribution function f(α). We do not propose which biological factors might determine the shape of this function. Instead, we temporarily represent the shape parametrically using *P.* After numerically investigating several possibilities, useful insights were obtained if the distribution is shaped like α^*P*−1^, with α between 0 and α_m_. The normalized distribution function is

This functional shape is quite simple; for example, if *P* = 1, the distribution is uniform; if *P* = 2, the distribution is triangular. Results for these special cases are given below.

Substituting Equation 3 into Equation 2,

The asymptotic time dependence of F(t) for any value of *P* can be revealed by a transformation of variables, u = αt. With this substitution, Equation 4 becomes

The integral was numerically evaluated and found to be a number of order 1. Thus, the asymptotic form for large values of t is a time-dependent power law, Equation 1 in the main text. A formula valid for any value of t and *P* can be derived from Equation 4 by expanding the exponential under the integral sign in a power series and integrating term-by-term,

Equation 6 was used to calculate the curves in Figure 7 when *P* is noninteger.

Closed-form formulas for F(t) can be found from Equation 4 for *P* = 1 and 2 as shown by Chen in a different context.^6^ They can also be derived from Equation 6.

As expected, these equations show that when α_m_t is large, the asymptotic form Equation 1 is recovered. They also trace the rollover of F(t) near t = 0; see Figure 7. The timescale where the rollover occurs is controlled by α_m_.

If the observations include no data in the rollover region near t = 0, Equation 1 can be used to determine the power-law parameters. If there is data near t = 0, Equation 6 can be used to determine the parameters (*P* and α_m_) by regression.
